# ESRP1 regulates alternative splicing of CARM1 to sensitize small cell lung cancer cells to chemotherapy by inhibiting TGF-β/Smad signaling

**DOI:** 10.18632/aging.202295

**Published:** 2021-01-20

**Authors:** Meng Zheng, Yuchun Niu, Junguo Bu, Shumei Liang, Zhilin Zhang, Jianhua Liu, Linlang Guo, Zhihua Zhang, Qiongyao Wang

**Affiliations:** 1 Department of Pathology, Zhujiang Hospital, Southern Medical University, Guangzhou, China; 2 Department of Respiratory Medicine, The First Affiliated Hospital of Hebei North University, Zhangjiakou, China; 3 Department of Oncology, Zhujiang Hospital, Southern Medical University, Guangzhou, China; 4 Department of Radiotherapy, Zhujiang Hospital, Southern Medical University, Guangzhou, China; 5 Department of Radiotherapy, The First Affiliated Hospital of Hebei North University, Zhangjiakou, China

**Keywords:** small cell lung cancer, chemoresistance, ESRP1, CARM1, TGF-β/Smad pathway

## Abstract

Epithelial splicing regulatory protein 1 (ESRP1) is an RNA-binding protein that regulates alternative splicing of mRNA. ESRP1 plays an important role in chemoresistance of various cancers, including breast cancer, colon cancer and non-small cell lung cancer. However, the role of ESRP1 and its mechanism in small cell lung cancer (SCLC) chemoresistance remains unclear. In this study, we found that ESRP1 is significantly downregulated in SCLC chemo-resistant cells compared with chemo-sensitive cells. Moreover, the expression of ESRP1 was significantly lower in SCLC tissues than that in normal adjacent tissues and positively correlated with overall survival. Overexpression of ESRP1 increased SCLC chemosensitivity, and induced cell apoptosis and cell cycle arrest, whereas knockdown of ESRP1 induced the opposite effects. ESRP1 could inhibit the growth of SCLC *in vivo*. Through mRNA transcriptome sequencing, we found that ESRP1 regulates coactivator-associated arginine methyltransferase 1 (CARM1) to produce two different transcripts CARM1FL and CARM1ΔE15 by alternative splicing. ESRP1 affects the chemoresistance of SCLC by changing the content of different transcripts of CARM1. Furthermore, CARM1 regulates arginine methylation of Smad7, activates the TGF-β/Smad pathway and induces epithelial-to-mesenchymal transition (EMT), thereby promoting SCLC chemoresistance. Collectively, our study firstly demonstrates that ESRP1 inhibits the TGF-β/Smad signaling pathway by regulating alternative splicing of CARM1, thereby reversing chemoresistance of SCLC. The splicing factor ESRP1 may serve as a new drug resistance marker molecule and a potential therapeutic target in SCLC patients.

## INTRODUCTION

Small cell lung cancer (SCLC) is a highly malignant tumor with rapid progression, strong invasiveness, early metastasis, and poor prognosis. At present, the main treatment for SCLC is chemotherapy with cisplatin combined with etoposide or irinotecan, which has not changed significantly in recent decades. Although patients with SCLC are very sensitive to chemotherapy in the early stages of treatment, most patients develop resistance to chemotherapy rapidly [1, 2]. Therefore, there is an urgent unmet need to find new strategies for the treatment of SCLC.

Alternative splicing (AS) is a regulatory method that follows RNA transcription. A gene can be translated to form multiple proteins with different functions after being regulated by alternative splicing [3]. Defects in alternative splicing often occur in various types of tumors due to mutations in the regulatory elements of specific cancer genes, or due to changes in the regulatory mechanism of splicing. RNA splicing factors have emerged as a new class of oncoproteins and tumor suppressors, which lead to disease progression by regulating RNA subtypes in landmark cancer pathways [4]. Therefore, dysregulation of alternative RNA splicing is the foundation of cancer and provides a potentially rich source for new therapeutic targets [5, 6]. Studies have reported that alternative splicing can significantly change the coding region of drug targets and play an important role in the treatment of tumor resistance [7]. For example, transforming growth factor-β (TGF-β)-induced AS of TAK1 promotes epithelial-mesenchymal transition (EMT) and drug resistance in cancer cells [8]. SRSF1 regulates the AS of caspase 9 and then influences the chemosensitivity of non-small cell lung cancer (NSCLC) [9]. AS of TRA2A promotes resistance to paclitaxel in triple-negative breast cancer [10]. Regulation of PTBP1 in AS of PKM promotes the drug resistance of pancreatic cancer cells to gemcitabine [11]. Therefore, these findings suggest that AS events may be biomarkers and potential therapeutic targets in tumors [12]. Epithelial splicing regulatory protein 1 (ESRP1) is a member of the hnRNP family of RNA binding proteins that regulate AS events associated with epithelial phenotypes [13]. ESRP1 participates in AS of important transcripts, such as FGFR2, CD44, p120-Catenin (CTNND1), and hMena (ENAH) in regulating the process of EMT involved in tumor metastasis [14]. In addition, ESRP1 can affect drug resistance in breast cancer and colon cancer [15, 16]. However, the functional role of ESRP1 in resistance to chemotherapy in SCLC remains unclear.

TGF-β plays an important role in cell proliferation and differentiation during development, and its deregulation is associated with numerous diseases [17]. The TGF-β signaling pathway works by activating the downstream effectors, like TGF-βII receptor and Smad3, leading to changes in cell phenotypes causing EMT and fibrosis [18, 19]. Abnormal activation of the TGF-β signaling pathway occurs frequently in tumors and results in chemoresistance of a variety of tumors, including SCLC, pancreatic cancer, colon cancer, and breast cancer [20–23]. In cancers, activated TGF-β ligands bind to TGF-β type II receptors and phosphorylate TGF-β type I receptors, which are an essential component of this bipartite transmembrane receptor; then phosphorylated TbRI subsequently phosphorylates intracellular Smad2 and Smad3 (Smad2/3). Activated Smad2/3 forms a complex with Smad4 and translocates to the nucleus, binding to specific DNA sequences, and regulating the transcription of target genes, such as Smad7 and Smad6. In a feedback loop, increased Smad7 blocks the TGF-β signaling pathway and reverses the TGF-β-induced downstream EMT, chemoresistance, proliferation, invasion, and migration [24]. The TGF-/Smad signaling pathway could be activated by different modifications. It has been reported that under the action of bone morphogenetic protein (BMP), protein arginine methyltransferase 1 (PRMT1) could methylate Smad6 and Smad7 on the BMP receptor complex, thereby promoting its separation from the BMP receptor, activating BMP-induced Smad1 and Smad5, and also phosphorylating Smad3 to promote TGF-β-induced EMT and epithelial stem cell generation [25]. Although our research group has previously found that Smad7 depletion enhanced SCLC chemoresistance [20], whether Smad7 is regulated by the PRMT family and promotes chemoresistance is unclear.

Our study for the first time demonstrated the inhibitory effect of ESRP1 on chemoresistance of SCLC through *in vivo* and *in vitro* experiments. Through mRNA transcriptome sequencing, we found that ESRP1 can regulate AS of CARM1 to produce two transcripts, the full-length transcript of CARM1 (CARM1FL) and the deletion of exon 15 transcripts (CARM1ΔE15). Furthermore, CARM1FL regulates arginine methylation of Smad7 and activates the TGF-β pathway, thereby promoting SCLC chemoresistance. Overall, our research provides new avenues for the treatment of patients with SCLC, and ESRP1 may be used as a new therapeutic target.

## RESULTS

### ESRP1 positively correlates with the survival of patients with SCLC

**Figure 1:**
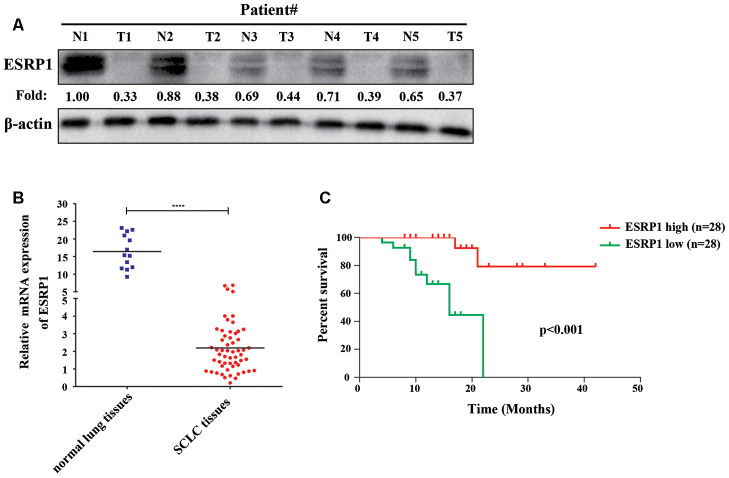
**ESRP1 positively correlates with overall survival.** (**A**) ESRP1 levels from five paired SCLC tumors (T) and normal (N) tissues were analyzed by western blotting. (**B**) ESRP1 mRNA levels in SCLC tissues and adjacent noncancerous lung tissues. (**C**) Kaplan–Meier analyses of the correlations between ESRP1 mRNA level and overall survival in SCLC patients. *, *p*<0.05; **, *p* <0.01; ***, *p* <0.001; ****, *p* <0.0001.

To clarify the role of ESRP1 in SCLC, we examined its clinical relevance in patients with SCLC. We first analyzed the differential expression of ESRP1 in the tumor and adjacent normal tissues from five patients by Western blot, which revealed that the expression of ESRP1 was significantly decreased in SCLC tumor tissues as compared with corresponding adjacent normal tissues (Figure 1A). Then, we investigated levels of ESRP1 mRNA expression in 56 human SCLC and 13 normal lung tissue samples by qRT-PCR. The results of this analysis revealed lower ESRP1 mRNA expression in SCLC tissues than in normal tissues (Figure 1B). Moreover, analysis of the results showed that low expression levels of ESRP1 was significantly associated with smoking history, extensive disease, and worse status in patients with SCLC (Table 1). Kaplan-Meier analysis revealed that low ESRP1 mRNA levels in SCLC tissues correlated with reduced overall survival (Figure 1C). The univariate analysis showed that ESRP1 expression levels correlated with disease stage and survival in SCLC, whereas the multivariate analysis suggested that ESRP1 was not an independent predictor of prognosis in these patients (Table 2). Overall, the above analysis indicated that ESRP1 was down-regulated in SCLC chemoresistant cells and tumor tissues, and that the expression of ESRP1 was correlated with the overall survival of SCLC patients.

**Table 1:** Clinical characteristics of 56 patients with SCLC according to the ESRP1 expression level.

**Variable**	**ESRP1**		***p* value**	
	**Low**	**High**		
Age, years, ≤62: >62	8:20	9:19		0.084
Sex, male: female	21:7	23:5		0.424
Smoking history, Yes: No	17:11	17:11		0.000*
Disease stage, LD: ED	15:13	15:13		0.000*
Status, Survival: Death	18:10	26:2		0.02*

Used χ^2^ test to test the correlation between two variables (*represents statistically significant differences (p < 0.05)).

LD limited-stage diseases, ED extensive-stage disease.

**Table 2:** Univariate and multivariate Cox-regression analysis of various prognostic parameters in patients with SCLC.

**Variable**	**Univariate analysis**			**Multivariate analysis**	
	***p***	**HR (95%CI)**		***p***	**HR (95%CI)**
ESRP1	0.02*	0.52(0.30-0.90)		0.193	0.59(0.27-1.30)
Age	0.766	0.99(0.90-1.08)		0.423	1.05(0.93-1.18)
Sex	0.417	1.79(0.44-7.30)		0.653	0.62(0.07-5.08)
Disease stage	0.002*	27.54(3.32-228.5)		0.016*	17.81(1.72-184.9)
Smoking history	0.155	2.78(0.68-11.34)		0.065	9.38(0.87-100.9)

(*represents statistically significant differences (p < 0.05)).

### ESRP1 is reduced in chemoresistant SCLC cells and inhibits chemoresistance of SCLC cells *in vitro* and *in vivo*

**Figure 2:**
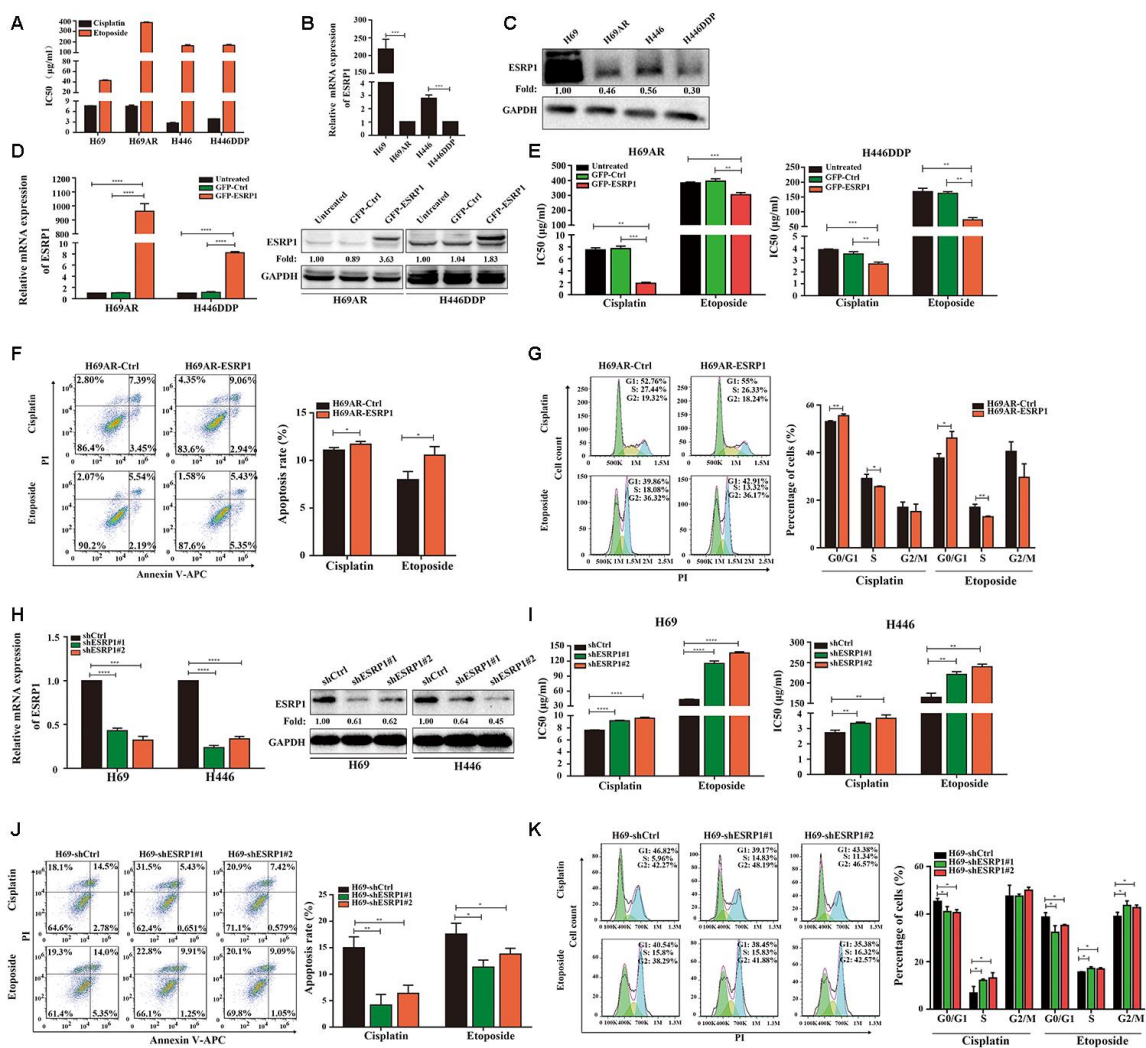
**ESRP1 is reduced in chemoresistant SCLC cells and inhibits chemoresistance of SCLC *in vitro*.** (**A**) IC_50_ values of cisplatin and etoposide in SCLC cells. (**B**) Differential expression of ESRP1 in SCLC chemoresistant cells (H69AR and H446DDP) and chemosensitive cells (H69 and H446) at transcriptional level. (**C**) Differential expression of ESRP1 in SCLC chemoresistant cells (H69AR and H446DDP) and chemosensitive cells (H69 and H446) at translational level. (**D**) Detection of ESRP1 up-regulation efficiency in H69AR and H446DDP cells at the transcriptional level and translational level. (**E**) CCK8 assay to detect IC_50_ value of cisplatin and etoposide after overexpressing ESRP1 in H69AR and H446DDP cells. (**F**) Cells apoptosis was analyzed by flow cytometry after treatment with cisplatin and etoposide for 24 hours in ESRP1 overexpressed cells. (**G**) Cells cycle arrest was analyzed by flow cytometry after treatment with cisplatin and etoposide for 24 hours in ESRP1overexpressed cells. (**H**) Detection of ESRP1 down-regulation efficiency in H69 and H446 cells at the transcriptional level and translational level. (**I**) CCK8 assay to detect IC_50_ value of cisplatin and etoposide after down-regulation of ESRP1 in H69 and H446 cells. (**J**) Cell apoptosis was analyzed by flow cytometry after treatment with cisplatin and etoposide for 24 hours in ESRP1-downregulated cells. (**K**) Cells cycle arrest was analyzed by flow cytometry after treatment with cisplatin and etoposide for 24 hours in ESRP1-downregulated cells. *, *p*<0.05; **, *p* <0.01; ***, *p* <0.001; ****, *p* <0.0001.

Previous research by our group showed that the expression of circular RNA cESRP1 in SCLC chemoresistant cells was significantly lower than that in chemosensitive cells and suggested that cESRP1 promotes chemosensitivity of SCLC by inhibiting the TGF-β signaling pathway [20]. Therefore, we speculated whether the ESRP1 protein formed by the same precursor RNA may also participate in the chemoresistance of SCLC. To confirm this hypothesis, we evaluated the mRNA and protein expression levels of ESRP1 in SCLC chemoresistant and chemosensitive cells. We found decreased expression of ESRP1 in H69AR and H446DDP cells compared with H69 and H446 cells, this result was in concordance with their higher IC_50_ values for chemotherapeutic drugs (Figure 2A–2C).

In order to clarify the role of ESRP1 in chemoresistance of SCLC, we transfected H69AR and H446DDP cells with lentivirus to stably overexpress ESRP1. The qRT-PCR and western blot assays were performed to judge vector transfection efficiency (Figure 2D). CCK8 assays were conducted to detect the chemosensitivity of SCLC cells to cisplatin and etoposide. The results showed that overexpression of ESRP1 increased chemosensitivity, with significant decreases in the IC_50_ values (Figure 2E). In addition, we also examined the effects of ESRP1 on cell apoptosis and cell cycle exposure to chemotherapeutic drugs. Compared with the control group, overexpression of ESRP1 significantly increased cell apoptosis and cell cycle arrest (Figure 2F, 2G).

Then we further verified the effect of ESRP1 on chemoresistance of SCLC in the parental sensitive H69 and H446 cells. We established stable ESRP1 knockdown in H69 and H446 cells, and through qRT-PCR and western blot experiments verified the knockdown efficiency of ESRP1 (Figure 2H). Contrary to the results of chemoresistant cells, decreased expression of ESRP1 resulted in promotion of SCLC chemoresistance (Figure 2I). Similarly, we confirmed the effect of ESRP1 knockdown on cell apoptosis and cell cycle. Contrary to the upregulation of ESRP1, the cell apoptosis and cell cycle arrest were reduced significantly (Figure 2J, 2K).

**Figure 3:**
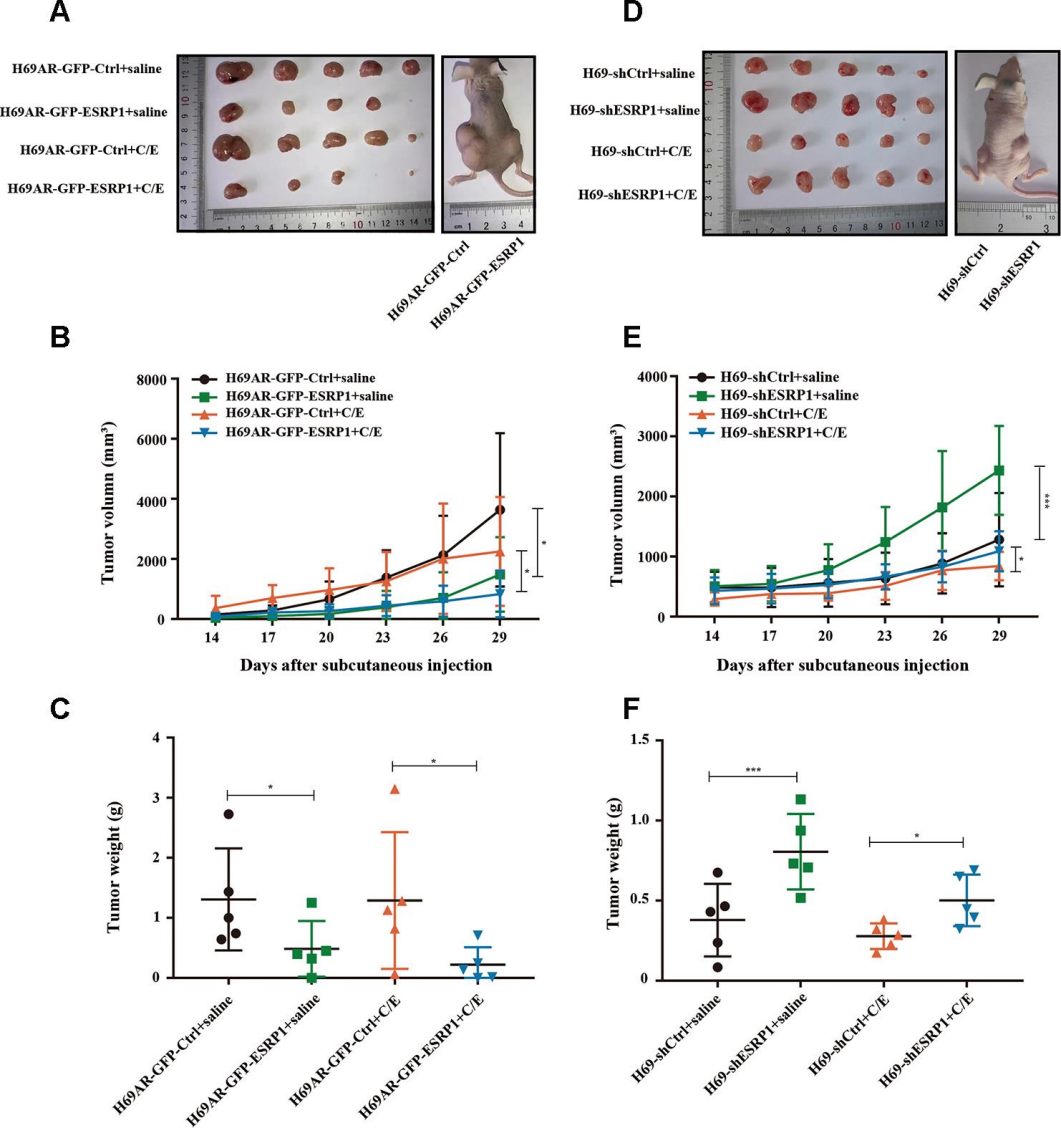
**ESRP1 inhibits the growth and chemoresistance of SCLC *in vivo*.** (**A**) Tumor formation of H69AR cells stably with up-regulated ESRP1 or the vector control (n = 5 nudes for each group). (**B**) Growth curve of tumor volumes. (**C**) Tumor weight taken from nude mice. (**D**) Tumors formation of H69 cells stably with down-regulated ESRP1 or the vector control (n = 5 nudes for each group). (**E**) Growth curve of tumor volumes. (**F**) Tumor weight taken from nude mice. *, *p*<0.05; ***, *p* <0.001.

To further assess whether ESRP1 influenced chemoresistance of SCLC *in vivo*, we subcutaneously transplanted H69AR and H69 with altered ESRP1 expression into nude mice (Figure 3A, 3D). Consistent with our *in vitro* findings, overexpression of ESRP1 significantly reduced tumor volume and weight compared with those of the paired control group (Figure 3B, 3C), whereas knockdown of ESRP1 led to the opposite results (Figure 3E, 3F). These results indicate that ESRP1 promotes chemosensitivity and inhibits the growth of SCLC *in vitro* and *in vivo*.

### ESRP1 mediates chemoresistance of SCLC by regulating alternative splicing of CARM1

**Figure 4:**
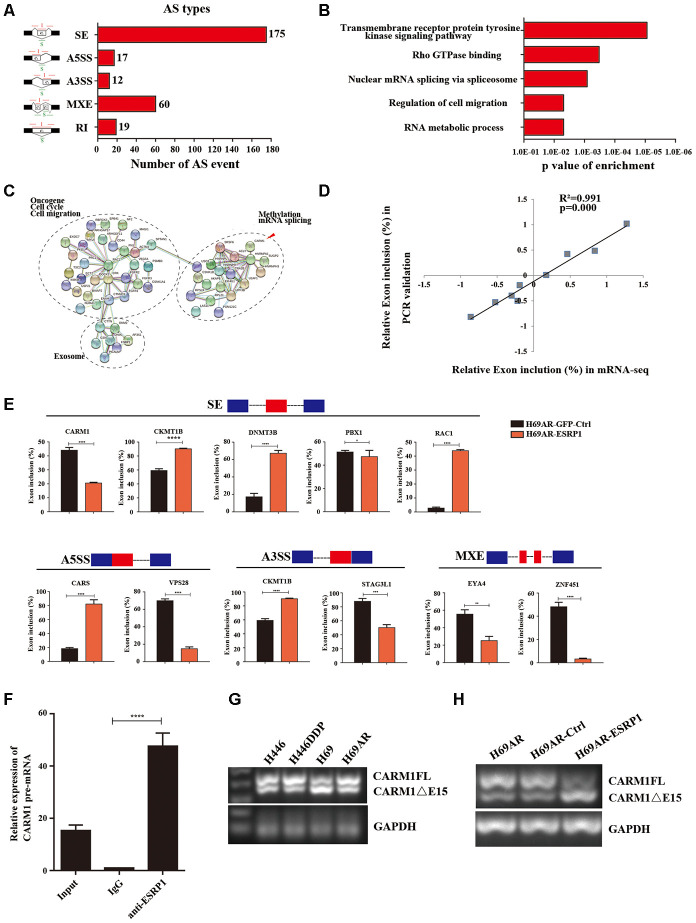
**Global regulation of the transcriptome by ESRP1 in SCLC chemoresistance-related genes.** (**A**) Quantification of the different AS events affected by ESRP1. (**B**) Gene ontology of ESRP1-regulated AS targets. Fisher exact p values were plotted for each enriched functional category. (**C**) Functional association network of ESRP1-regulated AS targets. The genes in (**C**) were analyzed using the STRING database, and subgroups are marked according to their functions. (**D**) Correlation between the relative changes in Exon inclusion ratio values observed by RNA-seq vs RT-PCR confirmation. (**E**) Validation of different types of ESRP1-regulated AS events by semiquantitative RT-PCR using H69AR cells transfected with ESRP1 or control vectors. The mean ± SD of Exon inclusion Ratio from three experiments were plotted. (**F**) The expression of CARM1 pre-mRNAs with ESRP1 was detected by RNA immunoprecipitation (RIP) assay in H69 cells. (**G**) Representative ethidium bromide stained agarose gel photo showing expression of CARM1FL and CARM1ΔE15 in chemoresistant and chemosensitive cells. (**H**) Representative ethidium bromide stained agarose gel photo showing expression of CARM1FL and CARM1ΔE15 after overexpressing ESRP1 in H69AR cells. *, *p*<0.05; **, *p* <0.01; ***, *p* <0.001; ****, *p* <0.0001.

To clarify the mechanism by which ESRP1 reversed SCLC chemoresistance, we conducted mRNA transcriptome sequencing with H69AR cells that stably overexpressed ESRP1. We identified 283 ESRP1-regulated AS events and found that various types of AS could be regulated by ESRP1, including skipped exon (SE), alternative 5′ss exon (A5SS), alternative 3′ss exon(A3SS), retained intron (RI), and mutually exclusive exons (MXEs) (Figure 4A and Supplementary Table 2). Then, we analyzed cellular functions of ESRP1-regulated AS events using gene ontology (GO) and found that ESRP1 affected genes in the RNA processing pathway, including mRNA splicing and mRNA metabolic processes (Figure 4B). Moreover, ESRP1 targets were also enriched with drug resistance-related functions, such as transmembrane receptor protein tyrosine kinase signaling pathway and Rho GTPase binding. Intriguingly, several ESRP1-regulated AS events were found to regulate the response to the chemical stimulus pathway. Although this enrichment of response to chemical stimulus was slightly below our significance cut-off, the changes in exon inclusion ratios were fairly large and, therefore, may have significant functional effects on consequences of drug resistance. Many of the ESRP1-regulated splicing targets were functionally connected to well-linked interaction networks, as judged by the Search Tool for the Retrieval of Interacting Genes/Proteins (STRING) (Figure 4C). As expected, a large subgroup of ESRP1 targets contained genes involved in RNA processing. Surprisingly, the other two subgroups included many genes involved in cell migration and cell cycle. Taken together, these results suggest that the biological processes affected by ESRP1 are related to apoptosis, proliferation, migration, and tumorigenesis. We subsequently validated mRNA-seq results by measuring splicing change in five newly identified targets that were selected arbitrarily to include genes with a drug resistance-related function (Figure 4E). We confirmed that ESRP1 either positively or negatively controlled all endogenous AS events tested and that the relative changes in exon inclusion ratio obtained from RT-PCR were highly consistent with mRNA-seq (Figure 4D). Taken together, these data imply that ESRP1 may be a key regulator that controls cancer progression.

We then explored the downstream splicing targets of ESRP1. Studies show that CARM1 methylated MED12 can increase chemosensitivity of breast cancer cells [26]. Wang et al. have confirmed that there are two major splice variants of CARM1 produced by AS, which are the full-length transcript of CARM1 (CARM1FL) and the deletion of exon 15 transcript (CARM1ΔE15). It has been confirmed that the deletion of exon 15 of CARM1 could block the self-methylation of CARM1, which in turn affects the transcriptional regulation of CARM1 on estrogen receptor-α (ERα). The CARM1 self-methylation site R511 is located on the fifteenth exon, and the self-methylation defect of CARM1 reduces the activity of CARM1 on the methylation of some protein substrates [27]. Therefore, we hypothesized that ESRP1 may regulate AS of CARM1 to affect SCLC chemoresistance. We performed a RIP assay using ESRP1 monoclonal antibody and found that ESRP1 enriched the pre-mRNA of CARM1 (Figure 4F). We detected the content of two splice isoforms of CARM1 in SCLC cells, and found that the content of CARM1FL in chemoresistant cells was significantly higher than that in chemosensitive cells, whereas the content of CARM1ΔE15 was completely opposite; this suggested that the alternative splicing of CARM1 is related to the chemoresistance of SCLC (Figure 4G). The RT-PCR results showed that ESRP1 increased the proportion of CARM1ΔE15 and decreased the proportion of CARM1FL (Figure 4H).

**Figure 5:**
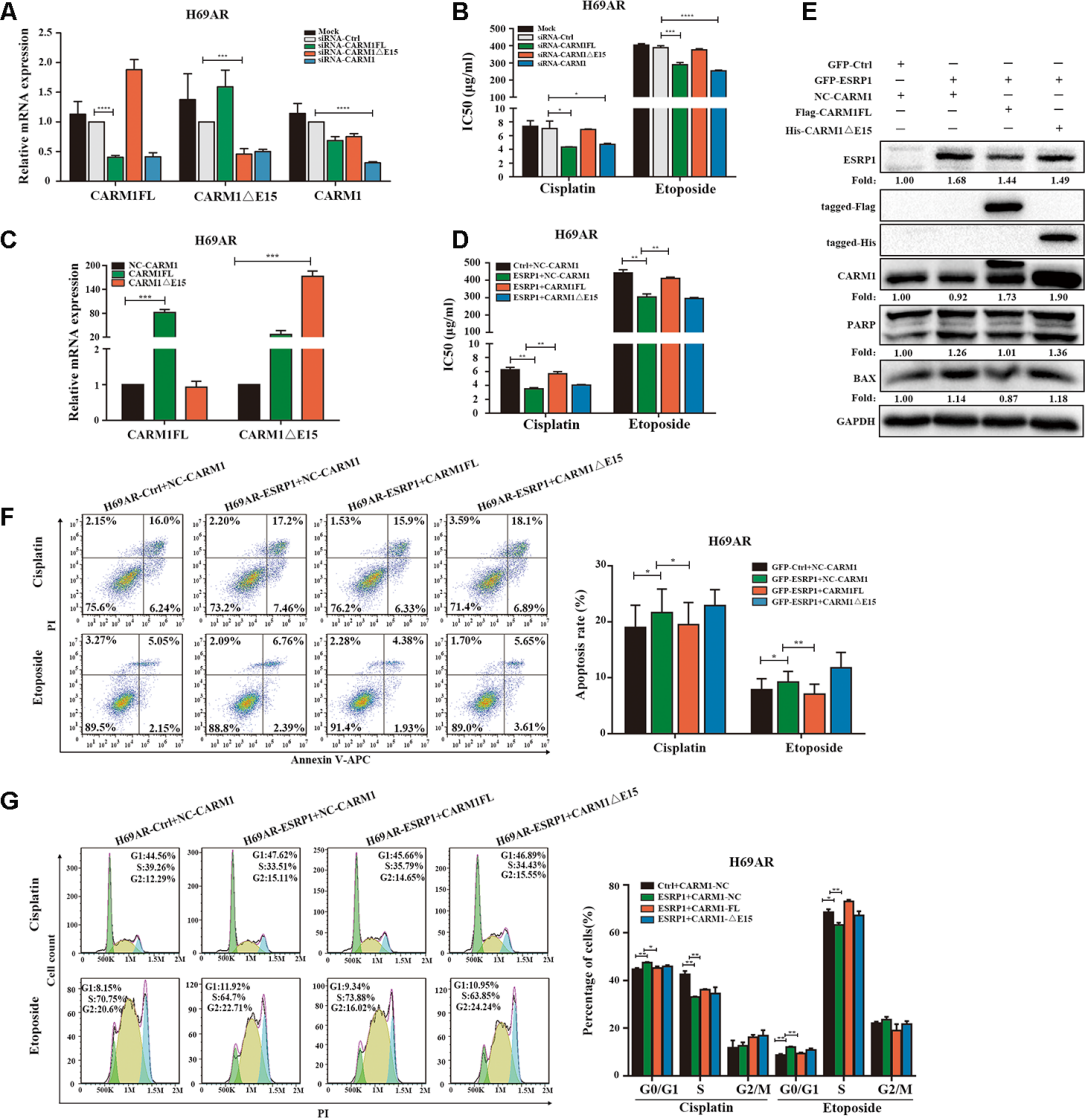
**ESRP1 mediates chemoresistance of SCLC by regulating alternative splicing of CARM1.** (**A**) Transfection of siRNA in H69AR cells and qRT-PCR assay to detect down-regulation efficiency. (**B**) Through CCK8 assay to detect IC50 value of cisplatin and etoposide after down-regulation of CARM1 in H69AR cells. (**C**) Transfected plasmids in H69AR and H446DDP cells to up-regulate the expression of CARM1FL and CARM1ΔE15, using qRT-PCR assay to detect down-regulation efficiency. (**D**) Through CCK8 assay to detect IC50 value of cisplatin and etoposide after up-regulation of CARM1 in H69AR cells. (**E**) Western blot assay was used to detect the expression of apoptosis-related proteins after H69AR cells were treated with cisplatin for 24 hours. (**F**) Cell apoptosis was analyzed by flow cytometry after H69AR cells were treated with cisplatin or etoposide for 24 hours. (**G**) Cell cycle arrest was analyzed by flow cytometry after H69AR cells were treated with cisplatin or etoposide for 24 hours. *, *p*<0.05; **, *p* <0.01; ***, *p* <0.001; ****, *p* <0.0001.

Since ESRP1 reduced the ratio of CARM1FL to CARM1ΔE15, in order to further examine the different roles of CARM1FL and CARM1ΔE15 in regulating chemoresistance of SCLC, we designed specific siRNAs targeting CARM1FL and CARM1ΔE15 transcripts respectively. We transfected siRNA in H69AR cells and verified the knockdown efficiency by qRT-PCR (Figure 5A). The CCK8 assay showed that the IC50 value was significantly decreased after CARM1FL or CARM1 knockdown, whereas the IC50 value did not change significantly after CARM1ΔE15 down-regulation (Figure 5B). These results suggested that knockdown of CARM1FL inhibits chemoresistance, CARM1ΔE15 may have no effect on chemoresistance of SCLC. To further demonstrate that ESRP1 affects chemoresistance of SCLC by regulating the content of different transcripts of CARM1, we performed rescue experiments in H69AR and H446DDP cells. We overexpressed CARM1FL or CARM1ΔE15 by using transfection plasmids and confirmed overexpression efficiency by qRT-PCR (Figure 5C and Supplementary Figure 1A). The CCK8 assays were performed in ESRP1-overexpressing cells with up-regulation of CARM1FL or CARM1ΔE15. The results showed significantly decreased IC50 values in ESRP1 overexpressing cells compared with the control group; upregulation of CARM1FL in ESRP1 overexpressing cells could reverse the decrease in IC50 values mediated by ESRP1 overexpression, whereas upregulation of CARM1ΔE15 cannot reverse the IC50 values (Figure 5D and Supplementary Figure 1B). Next, we conducted apoptosis and cell cycle cytometry analyses. We found that increased cell apoptosis and cell cycle arrest in ESRP1 overexpressing cells, and upregulation of CARM1FL in ESRP1 overexpressing cells could rescue the increase in cell apoptosis and cell cycle arrest regulated by ESRP1 overexpression. Similarly, upregulation of CARM1ΔE15 in ESRP1 overexpressing cells cannot rescue these (Figure 5F, 5G and Supplementary Figure 1C, 1D). In addition, western blot experiments showed that the changes to the expression levels of apoptotic proteins PARP and BAX are consistent with the results of flow cytometry (Figure 5E). These results demonstrate that ESRP1 affects chemoresistance of SCLC by reducing the proportion of CARM1FL, whereas CARM1ΔE15 has no effect on chemoresistance.

**Figure 6:**
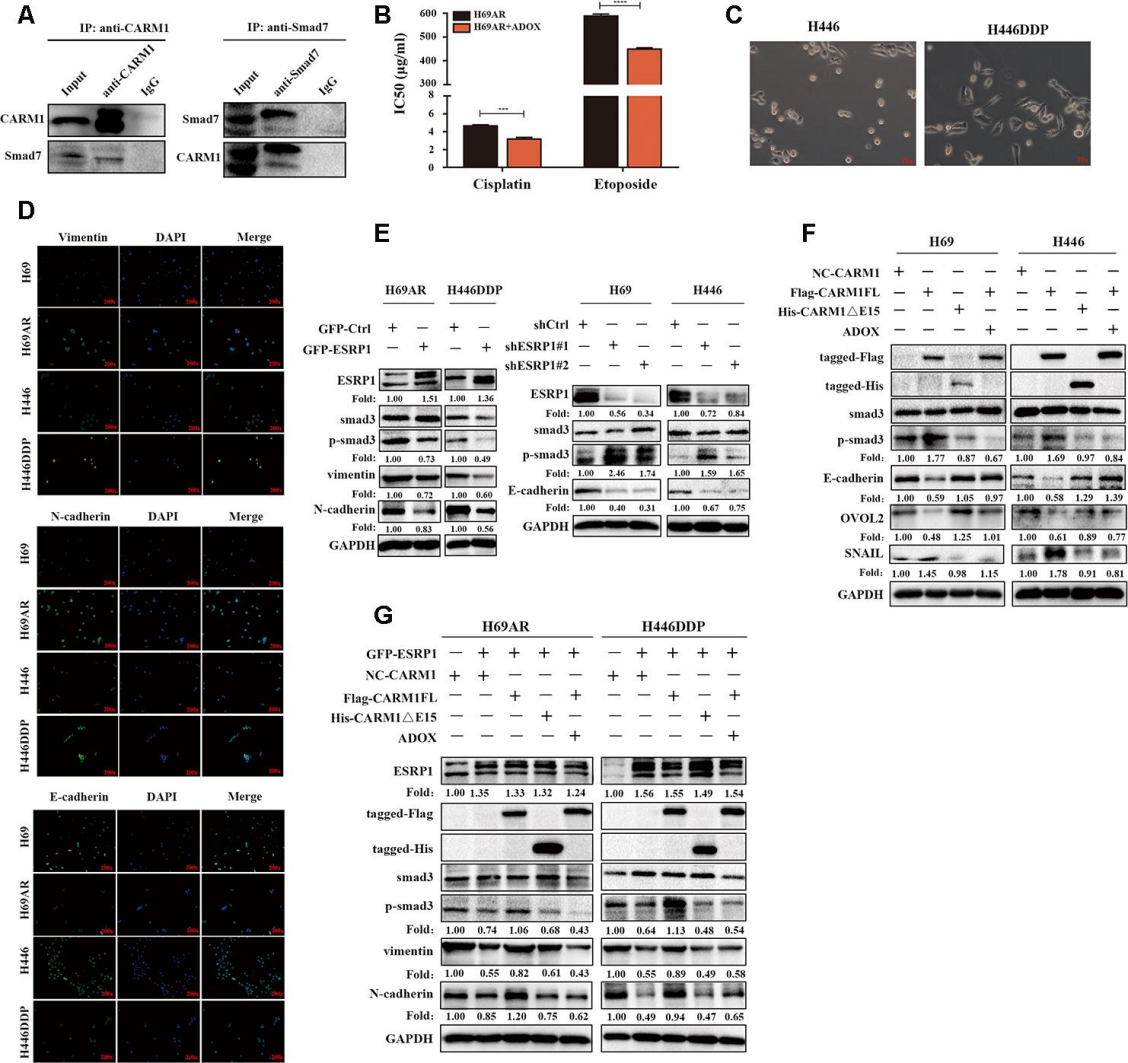
**CARM1 activates TGF-β/Smad signaling pathway by regulating arginine methylation of Smad7.** (**A**) Co-IP assays were conducted with specific CARM1 antibody and Smad7 antibody in H69AR cells. (**B**) H69AR cells were treated with 5 μM ADOX for 48 hours, then cells were exposed to cisplatin or etoposide for 24 hours, and IC50 values were measured by CCK8 assays. (**C**) Comparison of the basic morphology of chemosensitive cells and chemoresistant cells by optical microscope. (**D**) Immunofluorescence staining shows the expression of EMT marker protein. (**E**) The expression of ESRP1, Smad3, p-Smad3 and EMT-related proteins in stably upregulated or downregulated SCLC cells was detected by western blot assay. (**F**) Overexpressed CARM1FL or CARM1ΔE15 in H69 and H446 cells or treated cells with ADOX for 48 hours, western blot assay was performed to detect the expression of tag antibodies, Smad3, p-Smad3 and EMT-related proteins. (**G**) Overexpressed CARM1FL or CARM1ΔE15 in ESRP1-upregulated H69AR and H446DDP cells or treated cells with ADOX for 48 hours, western blot assay was performed to detect the expression of tag antibodies, Smad3, p-Smad3 and EMT-related proteins. *, *p*<0.05; **, *p* <0.01; ***, *p* <0.001; ****, *p* <0.0001.

CARM1 activates TGF-β/Smad signaling pathway by regulating Smad7 arginine methylation CARM1 belongs to the PRMT proteins family, PRMTs can methylate arginine residues in histones, epigenetically control the expression of a series of genes, and can also modify non-histone proteins, including signal-conducting systems, to regulate their functions [28, 29]. Now that CARM1 has been shown to be regulated by ESRP1 and affects chemoresistance in SCLC, then we need to explore the mechanism through which CARM1 works. We used the pMeS database to predict genes that may undergo arginine methylation and found that there were 17 arginine methylation sites in the sequence of Smad7 (Table 3). Research by Katsuno et al. found that PRMT1 can methylate Smad6 and Smad7, promote the separation of Smad6/Smad7 from the receptor, and activate BMP-induced Smad1/Smad5 and TGF-β-induced Smad2/Smad3 [25, 30]. Furthermore, our group’s previous research already has shown that Smad7 can participate in chemoresistance in SCLC [20]. Therefore, we speculated that CARM1 could regulate arginine methylation of Smad7 to activate the TGF-β/Smad signaling pathway thereby promoting the chemoresistance of SCLC. To confirm the relationship between CARM1 and Smad7, we performed a Co-IP assay in H69AR cells and found that CARM1 physically interacted with Smad7 (Figure 6A). Then we used methyltransferase inhibitor ADOX to treat H69AR cells and observed the IC50 values by CCK8 assay (Figure 6B). The results showed that the IC50 value significantly decreased after inhibiting the effect of methyltransferase, thereby suggesting that CARM1 indeed promoted SCLC chemoresistance by methylating Smad7.

**Table 3:** Prediction site of arginine methylation in Smad7 protein structure.

**Seq ID**	**R site**	**Peptides**	**Prediction score**
Sequence	3	XXXXXXXMFRTKRSALVRR	0.589355
Sequence	6	XXXXMFRTKRSALVRRLWR	0.640048
Sequence	11	FRTKRSALVRRLWRSRAPG	0.74468
Sequence	12	RTKRSALVRRLWRSRAPGG	0.921869
Sequence	15	RSALVRRLWRSRAPGGEDE	0.909561
Sequence	17	ALVRRLWRSRAPGGEDEEE	0.916009
Sequence	38	GGGGGGGELRGEGATDSRA	0.999324
Sequence	46	LRGEGATDSRAHGAGGGGP	0.99441
Sequence	57	HGAGGGGPGRAGCCLGKAV	0.99649
Sequence	67	AGCCLGKAVRGAKGHHHPH	0.973657
Sequence	118	ELLLQAVESRGGTRTACLL	0.9797
Sequence	122	QAVESRGGTRTACLLLPGR	0.953773
Sequence	131	RTACLLLPGRLDCRLGPGA	0.943454
Sequence	135	LLLPGRLDCRLGPGAPAGA	0.975753
Sequence	169	CKVFRWPDLRHSSEVKRLC	0.53692
Sequence	200	VCCNPHHLSRLCELESPPP	0.58066
sequence	213	LESPPPPYSRYPMDFLKPT	0.668651

In order to explore the relationship between chemoresistance and EMT phenotype, we first detected the basic morphology of chemoresistant cell H446DDP and chemosensitive cell H446. The results showed that H446 cells showed an epithelial phenotype, whereas H446DDP cells showed more of a mesenchymal phenotype (Figure 6C). Moreover, we analyzed the expression levels of EMT markers in chemoresistant cells and chemosensitive cells via immunofluorescence, we found that the expression levels of the mesenchymal markers vimentin and N-cadherin in chemoresistant cells H69AR and H446DDP were significantly higher than those of chemosensitive cells, whereas the expression level of epithelial marker E-cadherin was completely opposite (Figure 6D). We subsequently conducted rescue experiments to explore the mechanism by which the ESRP1/CARM1 axis involved in regulating the TGF-β/Smad signaling pathway and EMT. Overexpression of ESRP1 reduced the expression of phosphorylated-Smad3(p-Smad3), vimentin and N-cadherin; silencing of ESRP1 resulted in the opposite effects (Figure 6E). Overexpression of CARM1FL increased the expression of p-Smad3, EMT-inducing TF snail and reduced the expression of E-cadherin, EMT-inhibiting TF OVOL2, but the results could be rescued by addition of ADOX; Overexpression of CARM1ΔE15 had no effect on the protein expression (Figure 6F). Finally, we overexpressed ESRP1 in combination with upregulation of CARM1FL or CARM1ΔE15 in cells. We found that compared with overexpression of ESRP1 alone, simultaneous overexpression of ESRP1 and CARM1FL increased the expression of p-Smad3, vimentin, and N-cadherin. However, this effect was reversed after the addition of ADOX. Overexpression of ESRP1 and CARM1ΔE15 at the same time had no significant effects on the TGF-β/Smad signaling pathway and EMT compared with ESRP1 overexpression alone (Figure 6G). These results were consistent with previous functional experimental results, and proved that ESRP1 inhibits the activation of TGF-β/Smad signaling pathway by reducing the content of CARM1FL and thereby enhancing chemosensitivity of SCLC.

## DISCUSSION

Previous reports suggest that splicing factor can affect tumor chemoresistance in non-small cell lung cancer, breast cancer, prostate cancer, pancreatic cancer, and colon cancer [8, 11, 12, 31–36]. However, the function of splicing factor in SCLC is not clear. In this study, we firstly found that the expression of splicing factor ESRP1 was significantly decreased in SCLC chemoresistant cells and SCLC tissues and positively correlated with overall survival of patients. Therefore, we speculated that ESRP1 may be involved in the regulation of chemoresistance in SCLC. Overexpression of ESRP1 in SCLC cells induced cell apoptosis and cycle arrest, and increased SCLC chemosensitivity *in vitro* and *in vivo*. Downregulation of ESRP1 in SCLC cells led to the opposite results. To the best of our knowledge, this is the first report demonstrating that ESRP1 can reverse chemoresistance of SCLC, and ESRP1 may serve as a potential prognostic marker in patients with SCLC.

It has been reported that in tamoxifen-resistant breast cancer cells, knockout of ESRP1 could affect lipid metabolism and oxidoreductase processes, and increase cell basal and standby respiration capabilities, suggesting that ESRP1 may be a potential molecule for preventing drug resistance in ER+ breast cancer cells, analysis the role of ESRP1 in chemoresistance from glycolysis pathway [37]. To investigate the mechanism by which ESRP1 affects SCLC chemoresistance, we analyzed the AS events and potential targets regulated by ESRP1 through mRNA transcriptome sequencing and selected several cancer-related targets to analyze the relationship between their exon ratio and ESRP1. It was previously reported that CARM1 can be selectively spliced to produce two transcripts, CARM1FL and CARM1ΔE15. These two transcripts have different distributions in breast cancer cells and are related to the biological function of ERα [27, 38]. CARM1 regulates methylation of arginine residues in histones and non-histones involved in mRNA formation, transcriptional co-activation, cell proliferation and cell differentiation [28, 39]. It has been reported that CARM1 is overexpressed in multiple cancers and regulates chemoresistance of cancer cells through arginine methylation, such as in breast cancer and pancreatic cancer [26, 40]. Therefore, we hypothesized that ESRP1 regulates the selective splicing of CARM1 to participate in chemoresistance of SCLC. RIP assays have shown that ESRP1 can enrich CARM1 pre-mRNA. Overexpression of ESRP1 in SCLC cells resulted in increases in the proportion of CARM1ΔE15 transcripts, whereas the proportion of CARM1FL transcripts decreased. To the best of our knowledge, this is the first study to demonstrate that ESRP1 can directly regulate AS of CARM1. The subsequent rescue experiments proved that ESRP1 can reverse chemoresistance of SCLC by reducing the proportion of CARM1FL, whereas CARM1ΔE15 did not participate in the regulation of SCLC chemoresistance.

CARM1 works by arginine methylation of proteins, which can mediate BAF155 and MED12 methylation to affect breast cancer chemoresistance [26, 41]. Then, we predicted the potential arginine methylation regulation targets of CARM1 and found that there were a large number of arginine methylation regulation sites in Smad7. Smad7 is an important negative feedback regulator of the TGF-β/Smad signaling pathway and is involved in resistance to chemotherapy by blocking of the TGF-β/Smad signaling pathway in multiple tumors [21, 22, 42]. Previous studies have shown that ESRP1 can participate in dynamic changes in alternative splicing during EMT [43–45]. Furthermore, our group’s previous research has shown that Smad7 can participate in chemoresistance of SCLC [20]. Therefore, our study firstly demonstrated that the TGF-β/Smad signaling pathway was inhibited by arginine methylation of Smad7 was involved in regulation of SCLC chemoresistance. In this study, we found that CARM1 physically interacted with Smad7 through a Co-IP experiment. We used the methyltransferase inhibitor ADOX to inhibit the methylation of Smad7, resulting in the increase of SCLC chemoresistance. We subsequently demonstrated that overexpression of ESRP1 inhibited the activation of the TGF-β/Smad signaling pathway, whereas overexpression of CARM1FL could reactivate the TGF-β/Smad signaling pathway and EMT, but this reactivation was inhibited by ADOX [46]. These experiments demonstrate that ESRP1 regulates the differential transcript content of CARM1 through alternative splicing, which in turn affects the regulation of CARM1 on Smad7 methylation and ultimately affects the activation of the TGF-β / Smad pathway. Although we have demonstrated that CARM1 physically interacted with Smad7 through a Co-IP experiment, the specific binding sites of CARM1 and Smad7 protein structure and the specific ways to affect the methylation of smad7 are unclear. We plan to do more in-depth mechanistic research to explore this subsequently.

In summary, our research firstly proved that splicing factor ESRP1 is related to SCLC chemoresistance. ESRP1 regulated the ratio of CARM1FL to CARM1ΔE15 in SCLC cells by alternative splicing of CARM1. CARM1FL regulates Smad7 methylation to activate the TGF-β/Smad pathway and promote SCLC chemoresistance. Our study may provide new avenues for treatment of patients with SCLC, and ESRP1 may be valuable as a potential prognostic marker and therapeutic target in SCLC.

## MATERIALS AND METHODS

### Clinical samples

Fifty-six tumor tissue sections were collected from the First Affiliated Hospital of Hebei North University and the Minzu Hospital of Guangxi Zhuang Autonomous Region that had been approved for use by the ethics committee. RNA extraction was done from paraffin-embedded tissue samples using the formalin-fixed paraffin-embedded (FFPE) RNA kit (Gibco, Guangzhou, China) to assess ESRP1 gene expression lever.

### Cell culture and reagents

Human chemosensitive SCLC cell lines (H69 and H446) and chemoresistant cells (H69AR) were purchased from the American Type Culture Collection (ATCC, USA). Human chemoresistant cell line (H446DDP) was induced by continuous cisplatin treatment of H446 cells in our laboratory. All cells were cultured in RPMI1640 basal medium (Gibco, Guangzhou China) plus 10% calf serum at 37° C, in a constant temperature incubator, containing 5% CO_2_.

Adenosine dialdehyde (ADOX) was purchased from Selleck Chemicals (Houston, Texas, USA); ADOX is an adenosine analog that inhibits methyltransferase in cells. Cells were treated with 5 μM ADOX for 48 hours and then related experiments were performed.

### RNA isolation and real-time polymerase chain reaction (qRT-PCR)

Extraction of total RNA from cells and FFPE tissues was done using TRIzol (Invitrogen, USA) and RNeasy FFPE Kit (Qiagen). NanoDrop 2000 (Thermo) was used to detect the concentration of extracted RNA. Next, qRT-PCR was performed with an ABI Illumina instrument (Foster, USA) using SYBR Green (Tiangen). The sequences of all qPCR primers are shown in the Supplementary Materials (Supplementary Table 1).

### Western blot

Total protein was extracted from cells using RIPA buffer (Biyuntian, China). The concentration of the extracted protein was detected using a BCA protein quantification kit (Biyuntian, China). Equivalent amounts of cell protein lysates were electrophoresed on 10% SDS-polyacrylamide gels. The membrane was blocked with 5% bovine serum albumin (BSA) for 2 hours at room temperature and then incubated overnight with a primary antibody at 4° C. The membrane was incubated with horseradish peroxidase-conjugated anti-rabbit or anti-mouse secondary antibody (EarthOx, USA) for 1 hour. The immune complexes were detected by chemiluminescence (ECL).

### siRNA and plasmid transfection

We transiently transfected cells with siRNA or plasmid using Lipofectamine 3000 (Thermo Scientific), P-3000 (Thermo Scientific) and OPTI-MEM (Invitrogen) to knockdown or overexpress CARM1, according to the manufacturer’s instructions.

### Cell counting kit-8 assays (CCK-8)

SCLC cells were cultured in 96-well plates with 1–2×10^4^ cells per well. The cells were treated with cisplatin (Shandong) or etoposide (Jiangsu) for 24 hours, then 10 μL CCK8 reagent was added and absorbance was detected at 450 nm. According to the OD value measured, the 50% inhibitory concentration (IC_50_ value) of the chemotherapeutic drug was calculated.

### Tumor xenograft experiments

Animal experiments for this study were performed in accordance with the institutional guidelines of Guangdong Province and the Use Committee for Animal Care. Female BALB/c nude mice aged 3–4 weeks were purchased from the Experimental Animal Center of Southern Medical University (Guangdong, China). The cell suspension was adjusted to 1×10^7^ cells per 200 μL phosphate-buffered saline (PBS). 200 μL cell suspension was injected subcutaneously on the flank of each nude mouse separately. Fourteen days after subcutaneous tumor implantation, nude mice were injected intraperitoneally with chemotherapeutic drugs (cisplatin 2.5 mg/kg, day 1 and etoposide 4 mg/kg, days 1–3) or saline. The tumor volume of nude mice was measured every 3 days. Nude mice were sacrificed after two cycles of drug injection.

### Flow cytometry and cell cycle analysis

Cells were treated with cisplatin or etoposide for 24 hours and then collected for further assays. Cell apoptosis was detected using the Annexin V APC/PI apoptosis kit (BestBio, China). In the cell cycle experiment, cells were fixed with 75% ethanol for 4 hours, and then we used RNase (Solarbio) and PI (Sigma, USA) to detect the cell cycle. All samples were analyzed by the CytoFLEX flow cytometer (Beckman).

### Immunofluorescence assays

Cells were seeded on slides for 24 hours. The cells fixed with 4% paraformaldehyde for 10 min and permeabilized with 0.5% Triton X-100 for 10 min at room temperature. Then, the cells were rinsed in PBS three times for 5 min each time and incubated with primary antibody overnight at 4° C. After three washings with PBS, the cells were incubated with fluorescent secondary antibody at room temperature for 1 hours. Then, the cells incubated with DAPI for 10 min at 37° C. Finally, we added an anti-fluorescence quencher on the slide and images were acquired using a confocal microscope.

### Co-immunoprecipitation assay (Co-IP)

We performed Co-IP assay, according to the operating instructions of the Co-IP kit (Thermo Scientific). The immunoprecipitated proteins were detected by western blots.

### RNA immunoprecipitation assay (RIP)

We performed the RIP assay, according to the operating instructions of the Magna RIP RNA-Binding Protein Immunoprecipitation Kit (Millipore).

### Nucleic acid electrophoresis

Agarose (Biyuntian, China), 50× TAE buffer (Biyuntian), and 10000× Gel Red (Biyuntian) were used to prepare an electrophoresis solution. The nucleic acid which was amplified by qRT-PCR was mixed with 6× loading buffer (Biyuntian), and electrophoresis was performed at a constant voltage of 100 V in electrophoresis solution. A fluorescence picture was taken by electrophoresis.

### Statistical analysis

Statistical analysis of data was done using GraphPad Prism 7.0 and SPSS. Data are expressed as mean ± standard deviation (SD). Analysis of differences between data was done by two independent sample *t*-tests or one-way ANOVA. The association between ESRP1 expression and clinical features was analyzed by Chi-Square test or Fisher’s exact test. The survival curve was assessed by Kaplan-Meier analysis. *P*<0.05 was considered statistically significant.

## Supplementary Material

Supplementary Figure 1

Supplementary Table 1

Supplementary Table 2
