# Correction for: ESRP1 regulates alternative splicing of CARM1 to sensitize small cell lung cancer cells to chemotherapy by inhibiting TGF-β/Smad signaling

**DOI:** 10.18632/aging.206334

**Published:** 2025-10-31

**Authors:** Meng Zheng, Yuchun Niu, Junguo Bu, Shumei Liang, Zhilin Zhang, Jianhua Liu, Linlang Guo, Zhihua Zhang, Qiongyao Wang

**Affiliations:** 1 Department of Pathology, Zhujiang Hospital, Southern Medical University, Guangzhou, China; 2 Department of Respiratory Medicine, The First Affiliated Hospital of Hebei North University, Zhangjiakou, China; 3 Department of Oncology, Zhujiang Hospital, Southern Medical University, Guangzhou, China; 4 Department of Radiotherapy, Zhujiang Hospital, Southern Medical University, Guangzhou, China; 5 Department of Radiotherapy, The First Affiliated Hospital of Hebei North University, Zhangjiakou, China

**Keywords:** small cell lung cancer, chemoresistance, ESRP1, CARM1, TGF-β/Smad pathway

**This article has been corrected:** The authors found that in **Figure 4E**, the histograms for CKMT1B molecule are identical in both the “Skipped Exon (SE)” and “Alternative 3’ss Exon (A3SS)” alternative splicing types. However, CKMT1B should only be associated with the A3SS alternative splicing event. The authors have replaced the duplicate CKMT1B histogram in the SE alternative splicing type with DNMT3B data from the original experiments. The authors point out that CKMT1B was not selected for further study as a downstream signaling molecule of ESRP1 and this duplication does not affect the article’s conclusions.

The corrected **Figure 4** is presented below.

**Figure 4:**
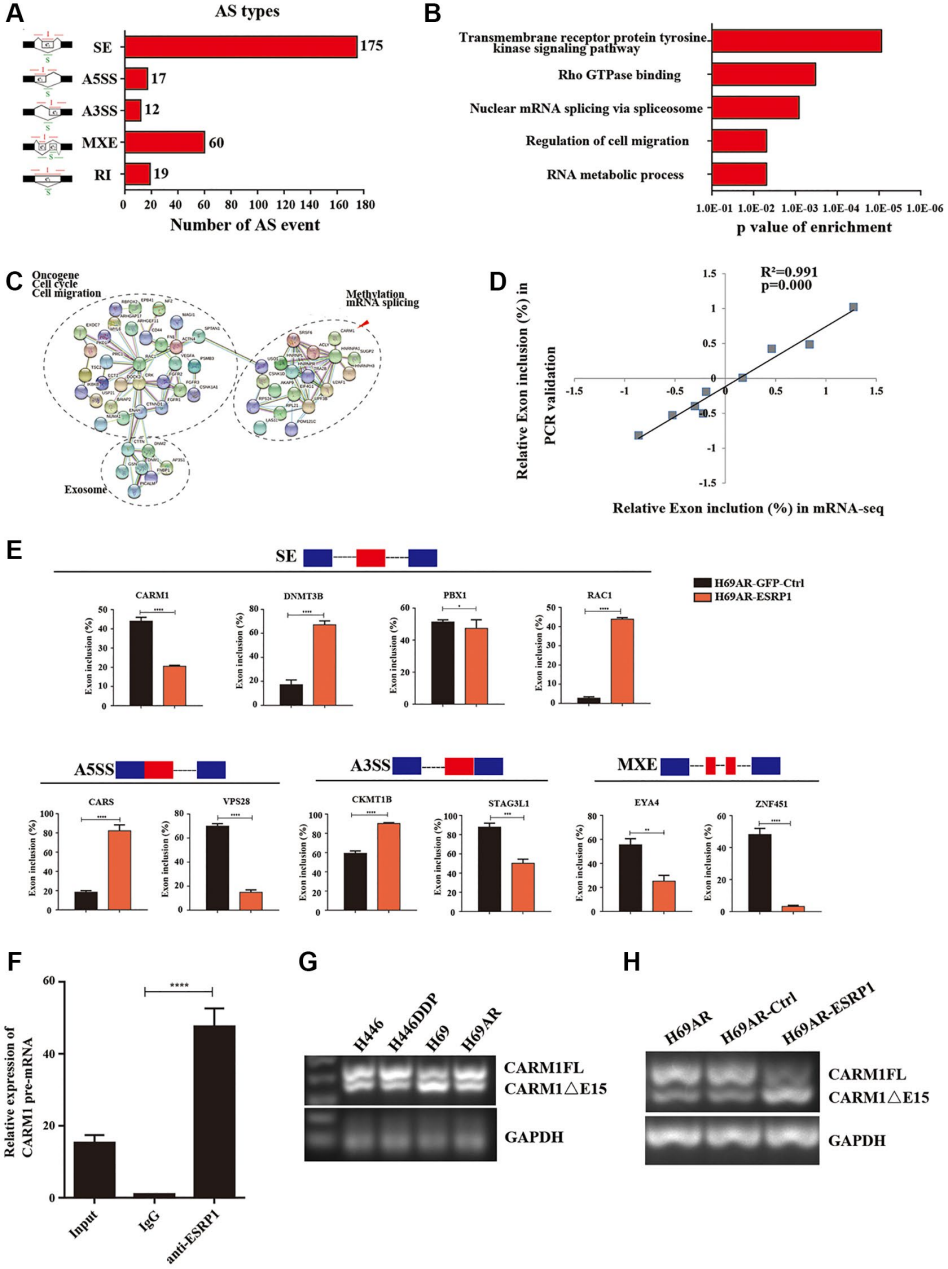
**Global regulation of the transcriptome by ESRP1 in SCLC chemoresistance-related genes.** (**A**) Quantification of the different AS events affected by ESRP1. (**B**) Gene ontology of ESRP1-regulated AS targets. Fisher exact p values were plotted for each enriched functional category. (**C**) Functional association network of ESRP1-regulated AS targets. The genes in (**C**) were analyzed using the STRING database, and subgroups are marked according to their functions. (**D**) Correlation between the relative changes in Exon inclusion ratio values observed by RNA-seq vs. RT-PCR confirmation. (**E**) Validation of different types of ESRP1-regulated AS events by semiquantitative RT-PCR using H69AR cells transfected with ESRP1 or control vectors. The mean ± SD of Exon inclusion Ratio from three experiments were plotted. (**F**) The expression of CARM1 pre-mRNAs with ESRP1 was detected by RNA immunoprecipitation (RIP) assay in H69 cells. (**G**) Representative ethidium bromide stained agarose gel photo showing expression of CARM1FL and CARM1ΔE15 in chemoresistant and chemosensitive cells. (**H**) Representative ethidium bromide stained agarose gel photo showing expression of CARM1FL and CARM1ΔE15 after overexpressing ESRP1 in H69AR cells. ^*^p < 0.05; ^**^p < 0.01; ^***^p < 0.001; ^****^p < 0.0001.

