# Supplementary material for: ESRP1 regulates alternative splicing of CARM1 to sensitize small cell lung cancer cells to chemotherapy by inhibiting TGF-β/Smad signaling
Source: Aging (Albany NY). 2021 Jan 20;13(3):3554–72. doi: 10.18632/aging.202295 (PMC7906186; doi:10.18632/aging.202295)
Supplement: Supplementary Figure 1 [file aging-13-202295-s001.pdf]

SUPPLEMENTARY FIGURE

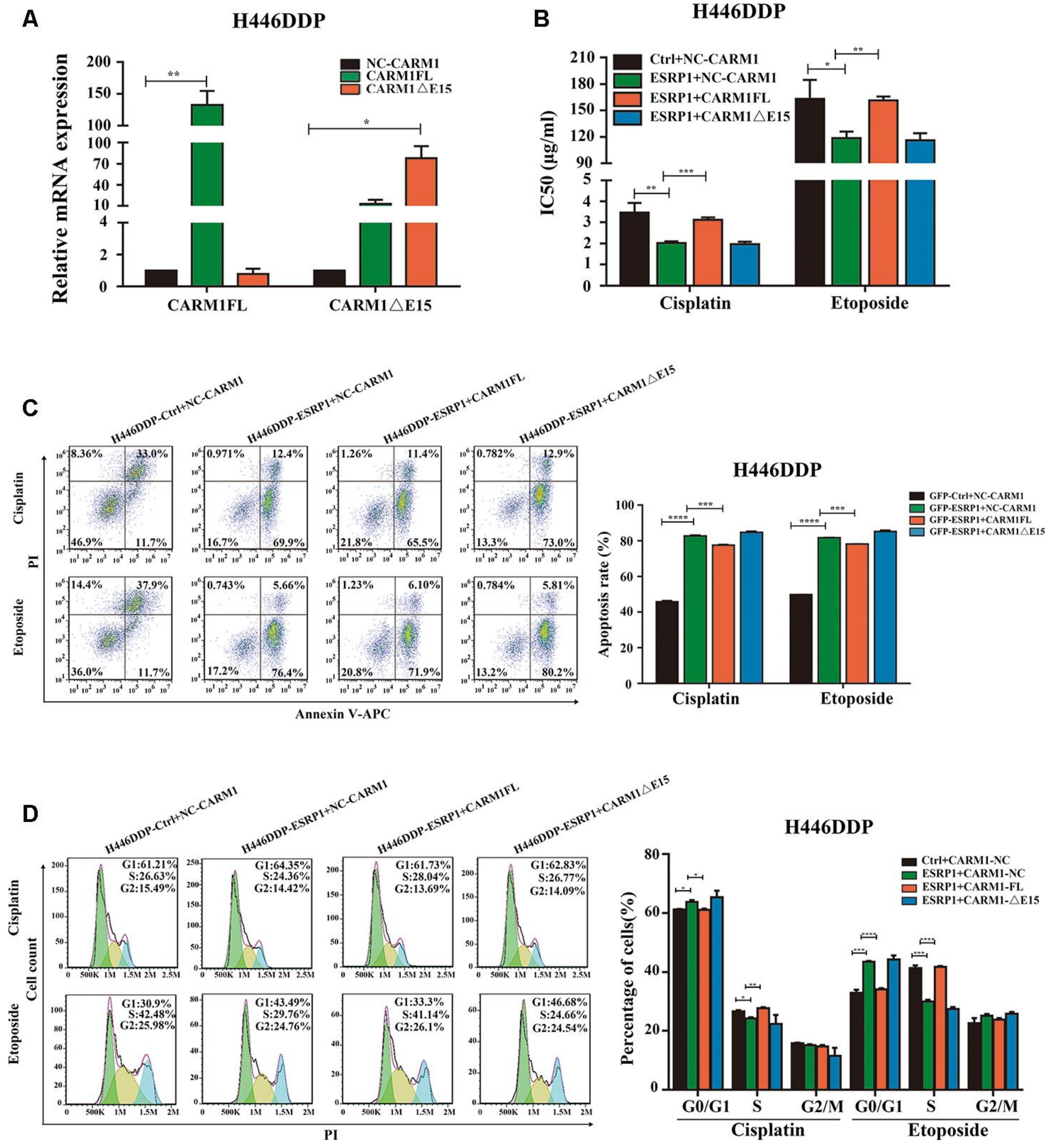

Supplementary Figure 1. ESRP1 mediates chemoresistance of SCLC by regulating alternative splicing of CARM1. (**A**) Transfected plasmids in H446DDP cells to up-regulate the expression of CARM1FL and CARM1ΔE15, using qRT-PCR assay to detect down-regulation efficiency. (**B**) Through CCK8 assay to detect IC50 value of cisplatin and etoposide after up-regulation of CARM1 in H446DDP cells. (**C**) Cell apoptosis was analyzed by flow cytometry after H446DDP cells were treated with cisplatin or etoposide for 24 hours. (**D**) Cell cycle arrest was analyzed by flow cytometry after H446DDP cells were treated with cisplatin or etoposide for 24 hours. *, *p*<0.05; **, *p* <0.01; ***, *p* <0.001; ****, *p* <0.0001.
