# Supplementary material for: ESRP1 regulates alternative splicing of CARM1 to sensitize small cell lung cancer cells to chemotherapy by inhibiting TGF-β/Smad signaling
Source: Aging (Albany NY). 2021 Jan 20;13(3):3554–72. doi: 10.18632/aging.202295 (PMC7906186; doi:10.18632/aging.202295)
Supplement: Supplementary Table 1 [file aging-13-202295-s002.pdf]

SUPPLEMENTARY TABLE

Supplementary Table 1. PCR primer sequences and siRNA sequences.

**qRT-PCR primer**
GARDH-Forward: ACAACTTTGGTATCGTGGAAGG
GARDH-Reverse: GCCATCACGCCACAGTTTC
ESRP1-Forward: TGCGTTGAGGAAGCATAAAG
ESRP1-Reverse: GGGTTGGAAGTGGAATGAGA
CARM1-Forward: ACGTTGCTTTCATCGGCTCC
CARM1-Reverse: CTTGGCGAACAGTGGTGACT
CARM1FL-Forward: TGTCAATCACACCCACTCCC
CARM1FL-Reverse: CTAGCTCCCGTAGTGCATGG
CARM1ΔE15-Forward: CACAACAACCTGATTCCTTTAGG
CARM1ΔE15-Reverse: TTGGTCGGGATGGACATGGG

**RT-PCR primer**
GARDH-Forward: ACAACTTTGGTATCGTGGAAGG
GARDH-Reverse: GCCATCACGCCACAGTTTC
CARM1-Forward: GATGCCGACCGCCTATGACT
CARM1-Reverse: GCGGGCAGGGACATCATTTG
RAC1-Forward: CAGCACGTGTTCCCGACATA
RAC1-Reverse: GAGGCATGGCAGGTGTAAGA
DNMT3B-Forward: GGAGTCTGCACGGGACCTAT
DNMT3B-Reverse: ATCTCCAAAGGGAGCACCGA
PBX1-Forward: CAGCATCATCCACCGCAAGT
PBX1-Reverse: GCTTCCATGGGCTGACACAT
CARS-Forward: CAGTTGCACGCAGAAGTCAG
CARS- Reverse: TCCAGCATGTTAGCAGTGGG
VPS28-Forward: CGTCCAGGTCTCAGTGCTGT
VPS28-Reverse: TTGTATTGGACCAGGAGCCG
CKMT1B-Forward: CCACGAGGAAGGCAGAGATT
CKMT1B-Reverse: GACTGGCCATGCAGTTGTTG
STAG3L1-Forward: GCAGCAAAACGACCACTGAA
STAG3L1-Reverse: CCTTCCAACTCCAAGTTTCCC
ZNF451-Forward: CCCTGAGGCATCTGAGTCAA
ZNF451-Reverse: GGTCCACACACAGTCTTGCT
EYA4-Forward: CAAGAGGGATGCCTGGCTAC
EYA4-Reverse: GGGCATGTTGTGCTAGTGCT

**siRNA sequence (5'-3')**
siRNA-CARM1FL: GGATTGTCAATCACACCCATT
siRNA-CARM1ΔE15: CUGAUUCCUUUAGGGUCCUTT
siRNA-CARM1: GGACAAGAUCGUUCUUGAUTT
shESRP1#1: GACAGCATTGCCCTATTAC
shESRP1#2: ATAAAGACTTGTTGGGTAA
